# The Effects of Mechanical Loading Variations on the Hypertrophic, Anti-Apoptotic, and Anti-Inflammatory Responses of Differentiated Cardiomyocyte-like H9C2 Cells

**DOI:** 10.3390/cells11030473

**Published:** 2022-01-29

**Authors:** Evangelos Zevolis, Anastassios Philippou, Athanasios Moustogiannis, Antonios Chatzigeorgiou, Michael Koutsilieris

**Affiliations:** Department of Physiology, Medical School, National and Kapodistrian University of Athens, 75 Micras Asias, Goudi-Athens, 115 27 Athens, Greece; ezevolis@med.uoa.gr (E.Z.); tfilipou@med.uoa.gr (A.P.); amoustog@med.uoa.gr (A.M.); achatzig@med.uoa.gr (A.C.)

**Keywords:** cardiomyocytes, cellular mechanotransduction, H9C2, mechanical stretch

## Abstract

Cardiomyocytes possess the ability to respond to mechanical stimuli by adapting their biological functions. This study investigated cellular and molecular events in cardiomyocyte-like H9C2 cells during differentiation as well as the signalling and gene expression responses of the differentiated cells under various mechanical stretching protocols in vitro. Immunofluorescence was used to monitor MyHC expression and structural changes during cardiomyoblast differentiation. Moreover, alterations in the expression of cardiac-specific markers, cell cycle regulatory factors, MRFs, hypertrophic, apoptotic, atrophy and inflammatory factors, as well as the activation of major intracellular signalling pathways were evaluated during differentiation and under mechanical stretching of the differentiated H9C2 cells. Compared to undifferentiated cells, advanced-differentiation cardiomyoblasts exhibited increased expression of cardiac-specific markers, MyHC, MRFs, and IGF-1 isoforms. Moreover, differentiated cells that underwent a low strain/frequency mechanical loading protocol of intermediate duration showed enhanced expression of MRFs and hypertrophic factors, along with a decreased expression of apoptotic, atrophy, and inflammatory factors compared to both high-strain/frequency loading protocols and to unloaded cells. These findings suggest that altering the strain and frequency of mechanical loading applied on differentiated H9C2 cardiomyoblasts can regulate their anabolic/survival program, with a low-strain/frequency stretching being, overall, most effective at inducing beneficial responses.

## 1. Introduction

H9C2 cardiomyoblasts are a cell line used as an alternative to cardiomyocytes. They are isolated from ventricular tissue and used in vitro as a mimetic model for cardiac muscle due to their biochemical, morphological, and hormonal signalling properties [1,2]. Thus, several studies have used H9C2 cells for investigating their differentiation features towards a cardiac-like phenotype [3]. Cardiomyocytes, as mechanosensitive cells, possess the ability to transduce mechanical stimuli to intracellular biochemical signals [4,5,6], while mechanotransduction mediates the adaptation mechanisms of these cells to mechanical loading [7].

Mechanosensitive extracellular and cellular elements have been shown to mediate the transduction of mechanical signals into the cell nucleus [8]. Through these mechanosensors, mechanical loading activates a complex network of signal transduction pathways that induce protein synthesis and increase the production of growth factors in mechanosensitive cells [9,10]. Moreover, mechanical stimuli modulate essential cellular processes, such as differentiation, survival, and apoptosis [11,12,13]. Under abnormal loading conditions, these processes can become maladaptive, altering the physiological function of cardiac muscle and leading to the development of pathological hypertrophy and heart failure [14,15,16,17,18,19,20]. Indeed, mechanical signals can affect cardiomyocyte differentiation in both physiological and pathological conditions, driven by multiple mechanotransduction pathways that coordinate the balance between protein synthesis and protein degradation, or muscle growth and atrophy [3,21].

Four transcription factors, the myogenic regulatory factors (MRFs) MyoD, Myf5, Myogenin, and MRF4, regulate myogenic differentiation [22], sharing the ability to convert various differentiated cell types to myogenic [23,24] and playing a similar role in cardiomyocyte-like cell myogenic differentiation [14,25,26]. Moreover, insulin-like growth factor-1 (IGF-1) signalling has been shown to play an important role in skeletal and cardiac cell growth through the activation of extracellular signal-regulated kinases (Erk 1/2) [25,26] as well as in the loading-induced adaptive cardiac hypertrophy through the activation of Akt [27,28], while potentially differential actions of IGF-1 isoforms in the myocardial repair/remodelling process and in cardiac myoblasts growth have been proposed [26].

On the other hand, mechanical loading can have beneficial or detrimental effects on differentiated cardiomyocytes, depending on its specific characteristics [29], by activating pro-inflammatory factors, such as IL-1β, TNF-α, IL-6, and NF-kB, as well as muscle atrophy and pro-apoptotic factors, such as myostatin, muscle-specific ubiquitin ligase Atrogin-1 (MaFbx), FoxO1, and p53, which have been negatively implicated in cell growth and survival, inducing cardiac muscle wasting and promoting heart failure [29,30,31,32,33,34,35,36].

The cellular and molecular phenotype of cardiomyocyte-like cells during their differentiation has not been fully characterized, while we have recently demonstrated that altering the features of mechanical stretching applied on advanced differentiation cardiomyoblasts induces different effects on their myogenic lineage [29]. More specifically, our previous findings emphasized that, for eliciting beneficial responses in the loaded cardiomyocytes, it is crucial to determine the optimal features of mechanical loading in terms of its loading/recovery characteristics.

In this study, we expand on our previous data to investigate the effects of more focused loading variations on signalling and expression responses, associated with the myogenic differentiation of these cells, by specifically varying the elongation and frequency of mechanical stretching. Thus, the aim of the present study was to further characterize the cellular and molecular signatures of cardiac myocyte-like H9C2 cells during their differentiation and to compare the hypertrophy/atrophy-, apoptosis-, and inflammation-related responses of the terminally differentiated cells to various mechanical loading protocols in vitro.

## 2. Materials and Methods

### 2.1. H9C2 Cell Culture

The H9C2 cell line of embryonic rat heart-derived ventricular cells was obtained from the American Type Culture Collection (ATCC, Manassas, VA, USA) and cultured as previously described [26]. Briefly, cells were grown in Dulbecco’s modified Eagle’s medium (DMEM) supplemented with 10% foetal bovine serum (FBS) and 1% penicillin/streptomycin at 37 °C in a humidified atmosphere of 5% CO_2_ in air, and the medium was changed every other day. The H9C2 cardiomyoblasts were seeded onto six-well flexible-bottomed culture plates coated with Collagen I (Flex I Culture Plates Collagen I; Flexcell International, Hillborough, NC, USA) and maintained in the growth medium until 70–80% confluence, then switched to a differentiation medium (2% horse serum, 1% of penicillin/streptomycin in DMEM). Cardiomyoblasts were allowed to differentiate into multinucleated myotubes for a five-day period during which the medium was changed every other day before their mechanical stretching, as described below.

### 2.2. Cardiomyocyte Mechanical Loading

Terminally differentiated H9C2 (myotubes) were stretched using the Flexcell FX-5000 strain unit (Flexcell International) that produces an isotropic two-dimensional (biaxial) strain of cells cultured on the flexible surface (silicone membrane) of the culture plates, as previously described [29]. Cardiomyotubes were subjected to four stretching protocols, in which the loading time was kept the same in each protocol, based on our previous findings [29], while the elongation and frequency of stretching varied: (a) 2% elongation (strain) at a frequency of 0.25 Hz; (b) 2% elongation at 1 Hz, (c) 12% elongation at 0.25 Hz, and (d) 12% elongation at 1 Hz. The waveform of the tension applied on the cardiomyocytes in the stretching cycle of each protocol mimicked the pressure fluctuations of a heartbeat in vivo.

### 2.3. Cell Lysis and RNA Extraction

Cell extracts were obtained by cell lysis using NucleoZOL (Mecherey-Nagel, Dueren, Germany). To further characterize the molecular phenotype of cardiomyocyte-like cells during their differentiation, cells were harvested and lysed on days 0, 3, and 5 of their differentiation. Stretched cardiomyotubes were harvested 12 h after the completion of the stretching protocol, while control (nonstretched) myotubes were also harvested 12 h after the end of each protocol. Total RNA was isolated from the lysates according to the manufacturer’s recommendations. The extracted RNA was dissolved in RNAase-free water (Invitrogen, Waltham, MA, USA) and the concentration and purity were determined spectrophotometrically (Thermo Nanodrop 2000,Thermo Scientific, Waltham, MA, USA) by absorption at 260 and 280 nm. Integrity of total RNA was confirmed by visual inspection of the electrophoretic pattern of 18S and 28S ribosomal RNA in ethidium bromide-stained 1% agarose gels under ultraviolet (UV) light. The total RNA samples were stored at −80 °C until further analysis for the determination of the mRNA levels of the genes of interest by reverse transcription and semi-quantitative real-time polymerase chain reaction (PCR) procedures.

### 2.4. Reverse Transcription and Real-Time PCR

Total RNA from each sample was used to produce single-stranded cDNA by reverse transcription using reverse transcriptase ProtoScript II (NEB, Ipswich, MA, USA) and the resultant cDNAs were utilized in real-time PCR, as described elsewhere [29,37]. The primer set sequences used for the specific detection of IGF-1 isoforms (IGF-1Ea, IGF-1Eb), myogenic regulatory factors (MyoD, Myogenin, MRF4), pro-apoptotic (FoxO1, FUCA, p53), atrophy (Atrogin-1, Myostatin), and pro-inflammatory (IL-1β, TNF-α, IL-6, NF-kB), and cell cycle regulation-related (Cyclin D1) factors, as well as cardiac-specific marker (Cardiac troponin type T) are shown in Table 1. To prevent detection of genomic DNA, the primer sets were designed to lie within different exons while, particularly, each set of primers for the detection of the IGF-1 isoforms was specific to detect only one IGF-1 transcript variant. To normalize the amount of total RNA present in each PCR reaction and the mRNA expression (relative quantification-dCt) of the genes of interest, glyceraldehyde 3-phosphate dehydrogenase (GAPDH) was used as a housekeeping gene (internal standard). Each sample was analysed in duplicate, and the resulting data were averaged. The specificity of the primers for the corresponding transcript was also confirmed by the melting curve analysis of samples, where there was only one melting curve for each sample, while electrophoretic analysis of the real-time PCR products further verified the specificity of the transcript of each gene of interest. Control for specificity included cDNA-free and template-free reactions.

### 2.5. Protein Extraction and Immunoblotting Analysis

Total proteins were extracted from H9C2 cardiomyoblasts as previously described [26,38]. Protein content was determined using a BCA protein assay kit (Thermo Scientific). Samples were stored in aliquots at –80 °C until Western blot analysis, as previously described [38]. Blots were incubated with the following primary antibodies for the immunodetection of Phospho-Akt, Phospho-Erk 1/2, Pospho-p38, Cardiac actin, MyoD, Myogenin and p53 proteins: rabbit monoclonal anti- Phospho-Akt (1:2000 dilution with 5% BSA in TBS-T) (4060; Cell Signaling, Danvers, MA, USA), anti-Phospho-p44/42 MAPK (Erk1/2; 1:2000 dilution with 5% BSA in TBS-T) (4370; Cell Signaling), rabbit monoclonal anti-Phospho-p38 (1:2000 dilution with 5% BSA in TBS-T) (#9211S; Cell Signaling), mouse monoclonal anti-Cardiac actin (1:2000 dilution with 5% BSA in TBS-T) (NBP2-67114; NOVUS Biologicals, Littleton, CO, USA), mouse monoclonal anti-MyoD (1:2000 dilution with 5% BSA in TBS-T) (sc-377460 Santa Cruz Biotechnology, Dallas, TX, USA), mouse monoclonal anti-Myogenin (1:2000 dilution with 5% BSA in TBS-T) (ab1835 Abcam, Cambridge, UK), and mouse monoclonal anti-p53 (1:2000 dilution with 5% BSA in TBS-T) (sc-126 Santa Cruz Biotechnology), respectively. A horseradish peroxidase-conjugated secondary anti-rabbit IgG (goat anti-rabbit, 1:2000 dilution; Santa Cruz Biotechnology), or anti-mouse IgG (goat anti-mouse, 1:2000 dilution; Santa Cruz Biotechnology) was used. The expected bands were visualized by exposure of the membranes to X-ray film after incubation with an enhanced chemiluminescent substrate for 3 min (ECL Supersignal west pico, Thermo Scientific). Anti-GAPDH antibody (1:2000 dilution; Santa Cruz Biotechnology) was used as an internal standard to correct for potential variations in the protein loading and to normalize the protein measurements on the same immunoblot. Band intensity was then semi-quantified using ImageJ software (NIH, Bethesda, MD, USA).

### 2.6. Immunofluorescence

H9C2 cardiomyocytes cultured on chamber slides were fixed, permeabilized, and stained using an indirect immunofluorescence method as previously described [34]. Briefly, cells were incubated with a primary mouse anti-Myosin Heavy Chain (MyHC) antibody (1:100, R&D, Minneapolis, MN, USA) and a goat anti-mouse IgG conjugated to the fluorescent Alexa 488 dye (1:2000, Abcam) secondary antibody, or Phaloidin conjugated with CF 488A (1:2000, Biotium, Fremont CA, USA), and after staining also with DAPI (1 µg/mL; 4083; Cell Signaling), they were viewed under a microscope (Olympus BX40; Olympus Corporation, Tokyo, Japan). In addition, ImageJ software [39] was used to perform morphological analyses of cardiomyoblasts H9C2 during their differentiation.

### 2.7. Cell Cycle Analysis

Flow cytometry was used to characterize the cell cycle profile of the H9C2 cardiomyoblasts. Undifferentiated cells were fixed in 70% ethanol overnight at 4 °C. The cells were then stained with DAPI (Cell Signaling) and cell cycle analysis was performed using a flow cytometer (Partec CyFlow, Gorlitz, Germany), where each phase of the cell cycle was calculated using the ModFit LT software Flow max 3.0 (Verity Software House, Topsham, ME, USA).

### 2.8. Statistical Analysis

One-way analysis of variance (ANOVA) with Dunn’s multiple comparison post hoc test or Student’s *t*-test was used for statistics, utilizing GraphPad Prism 5 (San Diego, CA, USA). All experiments were performed in triplicate and data are presented as mean ± standard error of the mean (SE). The level of statistical significance was set to *p* < 0.05.

## 3. Results

### 3.1. Differentiation Phenotype of Cardiomyocyte-like H9C2 Cells

#### 3.1.1. Cell Cycle and Morphological Analyses

**Figure 1:**
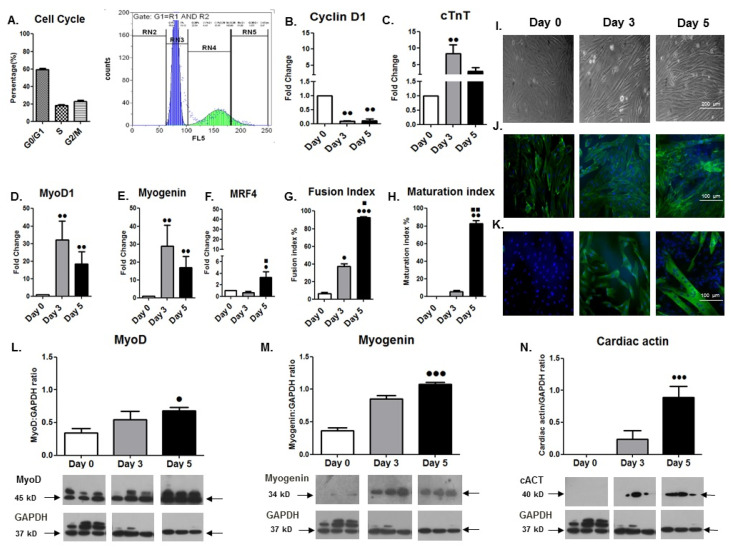
(**A**) Cell cycle progression of undifferentiated H9C2 cardiomyoblasts. Percentages of cells in each phase represent a normal cell cycle phenotype of H9C2 cardiomyoblasts. (**B**,**C**,**N**) Expression of cell cycle regulator Cyclin D1 and cardiac-specific markers during H9C2cardiomyoblast differentiation. Quantitative transcriptional analysis of Cyclin D1 revealed its downregulation during the differentiation process of cardiomyoblasts (**B**), along with the upregulation of the cardiac-specific markers troponin (**C**) and actin (**N**) (representative Western blot and immunoblotting quantification of cardiac actin). (**D**–**F**) Myogenic regulatory factors’ expression during cardiomyoblast differentiation. Quantitative analysis of the mRNA expression of muscle-specific transcription factors MyoD1, Myogenin, and MRF4 revealed a differential expression pattern, e.g., of early (MyoD1) vs. late (MRF4) myogenic factors in cardiomyoblasts during their differentiation. (**L**,**M**) Representative Western blots and immunoblotting quantification of MyoD (**L**) and Myogenin (**M**) protein expression are given. The mRNA expression values of each gene of interest have been normalized to the corresponding GAPDH mRNA and are expressed as fold changes. The expression of the proteins was also normalized to each corresponding GAPDH on the same immunoblot. The same blot has been used for GAPDH in (**L**–**N**). (**I**–**K**) Morphology-based analysis of H9C2 cardiomyoblasts during differentiation. Brightfield microscopy shows cardiomyoblast alterations over time during their differentiation process (**I**). Morphology alterations were further analysed using fluorescent DAPI, Phalloidin (**J**), and MyHC (**K**) immunostaining, documenting that these cells possess a myogenic differentiation potential. (**G**,**H**) Fusion and maturation index. Fusion index and (**G**) maturation index (**H**) values were calculated in cardiomyotubes immunostained with MyHC. Myotubes were considered differentiated cells that contained more than three nuclei. The fusion index was defined as the percentage of nuclei present in myotubes over the total number of nuclei present in the observed field, while the maturation index was defined as the percentage of nuclei present in myotubes that contained more than 10 nuclei over the total number of nuclei present in the observed field. Data were selected from 20 different and randomly chosen microscopic fields. Significantly different compared to day 0, ^•^ *p* < 0.05; ^••^ *p* < 0.01; ^•••^ *p* < 0.001. Significantly different compared to day 3, ^▪^ *p* < 0.05; ^▪▪^ *p* < 0.01. Mean + SE of three independent experiments performed in triplicate.

Analysis of the cell cycle phases distribution revealed a normal profile of cell cycle progression for the undifferentiated H9C2 cardiomyoblasts (Figure 1A). Moreover, morphology-based analysis during their differentiation showed a progressive differentiation process of the mononucleated cells towards their fusion and the formation of multinucleated cells (myotubes) (Figure 1I–K). Specifically, the H9C2 cardiomyocyte-like cells exhibited an increasing number of myotubes based on the cytoskeleton protein actin (phalloidin/DAPI staining), as well as the terminally differentiation marker MyHC staining (Figure 1J,K) from day 0 to day 5 of differentiation. The myogenic differentiation potential of these cells was further documented by the fusion index (FI) and the maturation index (MI) values over time, which were significantly higher at day 5 compared to day 3 or day 0 of differentiation (Figure 1G,H).

#### 3.1.2. Gene Expression Changes during Differentiation

**Figure 2:**
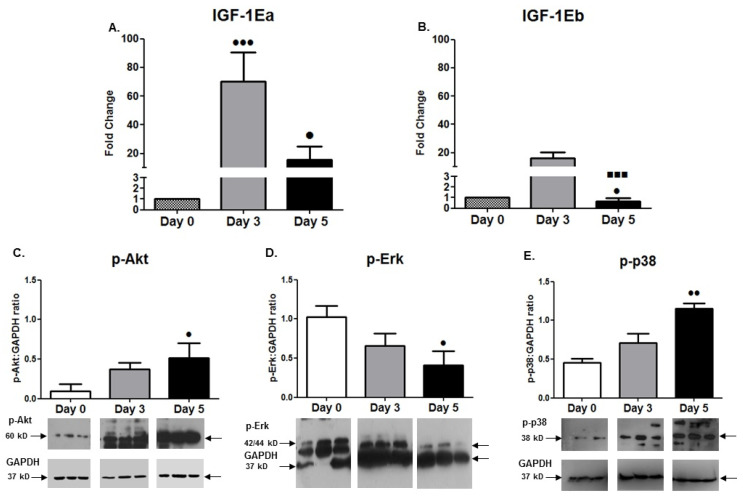
(**A**,**B**) IGF-1 isoforms’ expression during cardiomyoblasts differentiation. Quantitative analysis of IGF-1Ea and IGF-1Eb mRNA expression in cardiomyoblasts during their differentiation. (**C**–**E**) Alterations in the activation of the signalling proteins Akt, Erk1/2, and p38. Representative Western blots and immunoblotting quantification of p-Akt, p-Erk1/2, and p-p38phosphorylation in cardiomyoblasts during their differentiation process. The values of the phosphorylated proteins were normalized to each corresponding GAPDH on the same immunoblot. Significantly different compared to day 0, ^•^ *p* < 0.05; ^••^ *p* < 0.01; ^•••^ *p* < 0.001. Significantly different compared to day 3, ^▪▪▪^ *p* < 0.001. Mean + SE of three independent experiments performed in triplicate.

In accordance with the morphological alterations of the H9C2 cardiomyoblasts during their differentiation program, these cells exhibited a significant downregulation of Cyclin D1 expression over time, as expected, along with a gradual and significant increase in the cardiac-specific markers actin (Figure 1N) and troponin type T (Figure 1C), towards a cardiac-like phenotype. Moreover, changes in MRF expression were monitored during the differentiation of the H9C2 cardiomyoblasts, revealing a later peak of the late myogenic marker MRF4 (on day 5; Figure 1F) compared with the MyoD1 and Myogenin mRNA expression (on day 3; Figure 1D,E), while the protein levels of these two differentiation factors exhibited a time shift and peaked on day 5 of the myogenic differentiation, as expected (Figure 1L,M). In addition, differential expression of the IGF-1 isoforms, namely IGF-1Ea and IGF-1Eb, was shown for the first time during the H9C2 cardiomyoblasts’ differentiation, with a significant downregulation of IGF-1Eb at the advanced stage of differentiation (day 5) and a persistent upregulation of IGF-IEa up to day 5 of differentiation (Figure 2A,B).

#### 3.1.3. Changes in Signalling Pathways Activation during Differentiation

Along with the targeted gene expression responses during cardiomyocyte-like H9C2 cell differentiation, we also characterized the alterations in activation of major signalling pathways that regulate muscle cell growth, proliferation and differentiation [28]. Interestingly, we found a similar activation pattern of gradual increase in the phosphorylation of the important signalling mediators Akt and p38 over time, along with a gradual decrease in Erk1/2 activation (Figure 2C–E), with these alterations reaching significance (*p* < 0.05–0.01) at the advanced stage of cardiomyoblast differentiation (day 5).

### 3.2. Responses of Differentiated Cardiomyocyte-like H9C2 Cells to Various Mechanical Loading Protocols

#### 3.2.1. Myogenic Regulatory Factors

**Figure 3:**
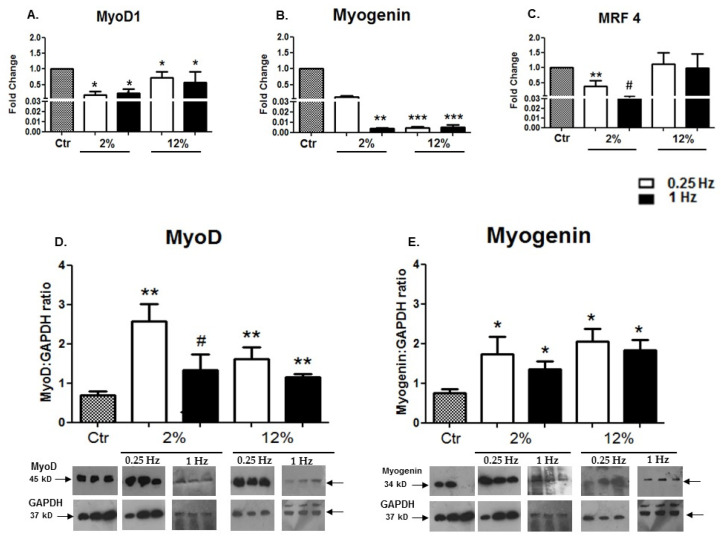
(**A**–**C**) Effects of cyclic mechanical loading on the expression of myogenic regulatory factors (MRFs). Quantitative analysis of transcriptional responses of (**A**) MyoD1, (**B**) Myogenin, and (**C**) MRF4 in differentiated cardiomyocytes subjected to different mechanical stretching protocols compared to the control (Ctr; nonstretched myotubes). (**D**,**E**) The mRNA values of MRFs in stretched myotubes have been normalized to the corresponding GAPDH mRNA and are expressed as fold changes compared to the control. Representative Western blots and immunoblotting quantification of MyoD (**D**) and Myogenin (**E**) subjected to the various mechanical loading conditions compared to the control; the proteins of interest were normalized to each corresponding GAPDH on the same immunoblot. The same blots have been used for GAPDH in Figure 3D,E, Figure 5C, and Figure 7A,B, except from the blot of GAPDH in panel A for the 2%/1 Hz stretching protocol. Significantly different compared to the control, * *p* < 0.05; ** *p* < 0.01; *** *p* < 0.001. Significantly different compared to the 2%/0.25 Hz stretching protocol, ^#^ *p* < 0.05. Mean + SE of three independent experiments performed in triplicate.

The expression levels of both early (MyoD) and late (Myogenin, MRF4) differentiation factors were examined in differentiated cardiomyoblasts to investigate the potential effects of mechanical loading on their myogenic lineage. It was revealed that, at the transcriptional level 12 h after the completion of the loading protocols, the stretching of cardiomyotubes induced the downregulation of those MRFs compared to the control (no stretch), regardless of the mechanical loading features (Figure 3A–C). At the protein level, mechanical loading led to increased expression of both MyoD and Myogenin compared to the control condition, potentially implying that the time point of 12 h after the completion of stretching does not correspond to the same “window” of transcriptional and translational responses (Figure 3D,E).

#### 3.2.2. Muscle Hypertrophy/Atrophy Factors

**Figure 4:**
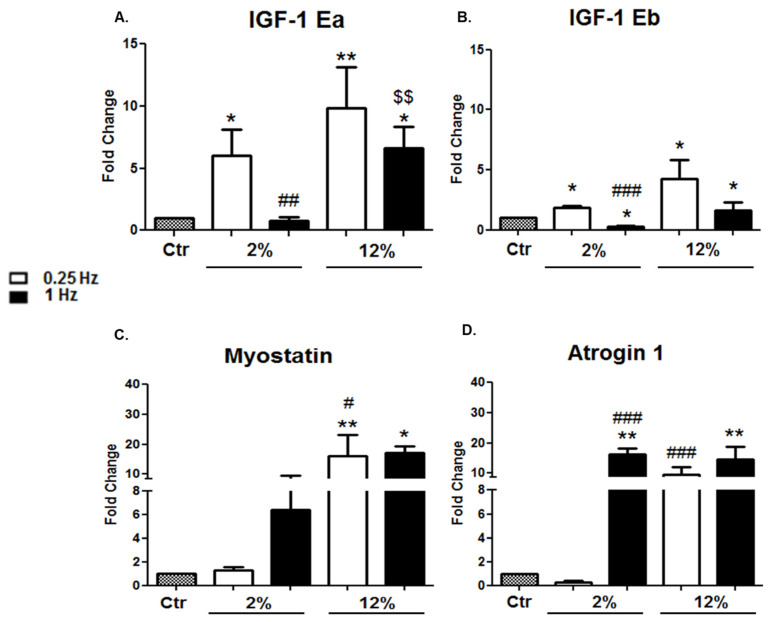
(**A**,**B**) Effects of mechanical stretching on the expression of the IGF-1 isoforms. Quantitative analysis of (**A**) IGF-1Ea and (**B**) IGF-1Eb mRNA levels in differentiated cardiomyocytes subjected to different mechanical stretching protocols compared to the control (Ctr; nonstretched myotubes). The transcriptional responses of IGF-1 isoforms in stretched cardiomyocytes have been normalized to the corresponding GAPDH values and are expressed as fold changes compared to the control. (**C**,**D**) Effects of mechanical stretch on the expression of muscle atrophy factors. Quantitative analysis of (**C**) Myostatin and (**D**) Atrogin 1 mRNA expression in differentiated cardiomyocytes subjected to the various mechanical loading conditions compared to the control (nonstretched myotubes); The mRNA values of the genes of interest in stretched myotubes have been normalized to the corresponding GAPDH mRNA and are expressed as fold changes compared to the control. Significantly different compared to the control, * *p* < 0.05; ** *p* < 0.01. Significantly different compared to the 2%/0.25 Hz stretching protocol, ^#^ *p* < 0.05; ^##^ *p* < 0.01; ^###^ *p* < 0.001. Significantly different compared to the 12%/ 0.25 Hz stretching protocol, ^$$^ *p* < 0.01. Mean + SE of three independent experiments performed in triplicate.

The effects of different stretching protocols on the expression of muscle hypertrophy and atrophy factors were also examined in differentiated cardiomyocytes. A similar expression profile was revealed for both IGF-1 isoforms regarding their responses to the different mechanical loading protocols. Interestingly, we found that the stretching frequency appears to regulate the expression pattern of IGF-1 isoforms, while the elongation affects the magnitude of their expression. Specifically, a low frequency (0.25 Hz) resulted in more pronounced increases in the expression of both IGF-1 isoforms compared to the control and high frequency (1 Hz), regardless of the elongation of stretching, while the higher the elongation applied (12%), the higher the expression of IGF-1 isoforms in each frequency tested. Interestingly, mechanical loading of high frequency (1 Hz)/low elongation (2%) appears to downregulate the expression of both IGF-1 isoforms (Figure 4A,B) and, conversely, to upregulate the cardiac and skeletal muscle atrophy factors Atrogin-1 and Myostatin (Figure 4C,D). Moreover, stretching of differentiated cardiomyoblasts seemed, overall, to upregulate Atrogin-1 and Myostatin compared to the control condition, except for the low-frequency (0.25 Hz)/low-elongation (2%) protocol, while high-elongation stretching was found to be a determinant of the upregulation of both atrophy factors at a low frequency (Figure 4C,D).

#### 3.2.3. Pro-Apoptotic Factors

**Figure 5:**
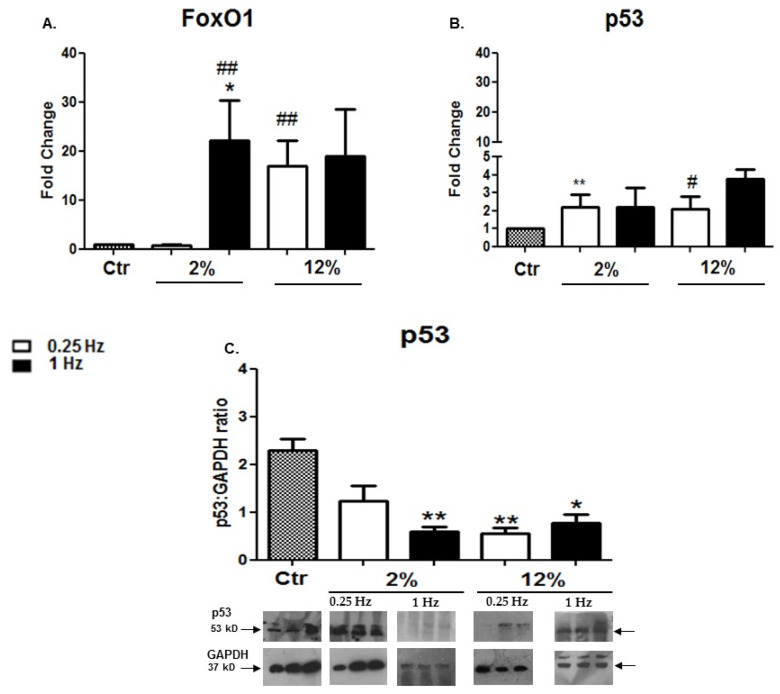
(**A**,**B**) Effects of mechanical loading on the expression of pro-apoptotic factors. Quantitative analysis of (**A**) FoxO1 and (**B**) p53 mRNA expression in differentiated cardiomyocytes subjected to various mechanical stretching protocols compared to the control (Ctr; nonstretched myotubes); the transcriptional responses of pro-apoptotic factors in stretched myotubes have been normalized to the corresponding GAPDH mRNA values and are expressed as fold changes compared to the control. (**C**) Representative Western blots and immunoblotting quantification of p53 subjected to the different stretching protocols compared to the control (nonstretched myotubes). The p53 protein levels were normalized to each corresponding GAPDH on the same immunoblot. The same blots have been used for GAPDH in Figure 3D,E, Figure 5C, and Figure 7A,B, except from the blot of GAPDH in panel A for the 2%/1 Hz stretching protocol, Significantly different compared to the control, * *p* < 0.05; ** *p* < 0.01. Significantly different compared to the 2%/0.25 Hz stretching protocol, ^#^ *p* < 0.05; ^##^ *p* < 0.01. Mean + SE of three independent experiments performed in triplicate.

Along with the loading-induced regulation of muscle hypertrophy/atrophy genes, the responses of apoptosis-related factors to mechanical loading were investigated in H9C2 cardiomyotubes. It was shown that FoxO1 exhibited an mRNA expression pattern similar to that of the atrophy factors Atrogin-1 and Myostatin, in response to the various stretching protocols. In particular, the high-frequency (1 Hz)/low-elongation (2%) protocol resulted in the significant upregulation of FoxO1 compared to the control while, as also observed in the atrophy genes, high-elongation stretching was found to be the determinant for the upregulation of FoxO1 at a low-frequency loading (Figure 5A; see also the responses of NF-kB below). In contrast with the FoxO1 responses, the low-frequency (0.25 Hz)/low-elongation (2%) protocol resulted in the transcriptional upregulation of p53 compared to the control condition (Figure 5B), while at the protein level the various stretching protocols were found to downregulate the expression of p53 12 h after the completion of stretching (Figure 5C) (see also the transcriptional vs. translational responses of MyoD and Myogenin above).

#### 3.2.4. Inflammation-Related Factors

**Figure 6:**
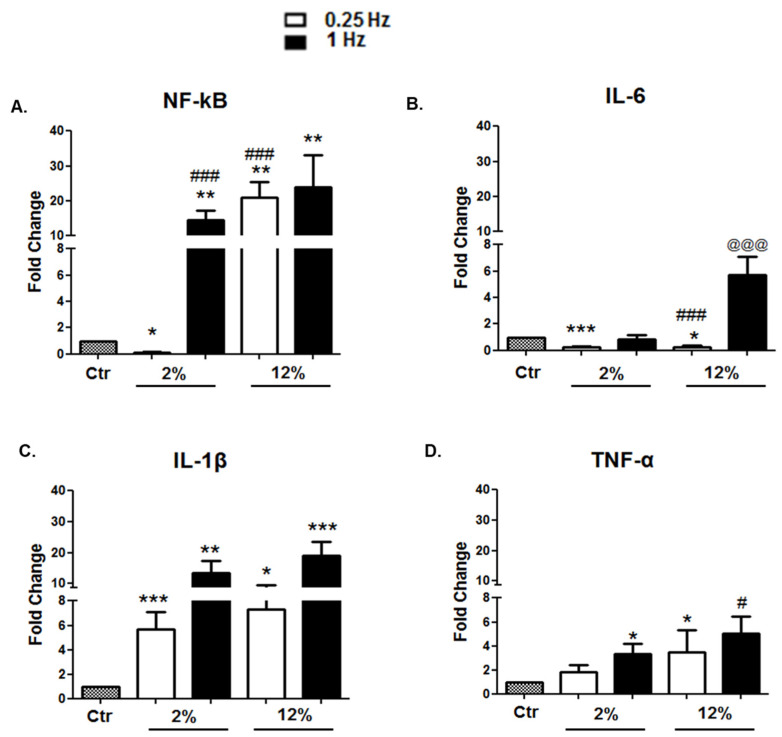
(**A**–**D**) Effects of mechanical loading on the expression of pro-inflammatory factors. Quantitative analysis of (**A**) NF-kB, (**B**) IL-6, (**C**) IL-1β, and (**D**) TNF-α transcriptional responses of differentiated cardiomyocytes subjected to various mechanical stretching protocols compared to the control (Ctr; nonstretched myotubes). The mRNA values of inflammation-related factors in stretched myotubes have been normalized to the corresponding GAPDH mRNA and are expressed as fold changes compared to the control. Significantly different compared to the control, * *p* < 0.05; ** *p* < 0.01; *** *p* < 0.001. Significantly different compared to 2%/0.25 Hz stretching protocol, ^#^ *p* < 0.05; ^###^ *p* < 0.001. Significantly different compared to the 12%/0.25 Hz stretching protocol, ^@@@^ *p* < 0.001. Mean + SE of three independent experiments performed in triplicate.

In parallel with the effects of mechanical loading on the expression of myogenic, hypertrophy/atrophy, and apoptosis-related factors, we examined the effects of mechanical loading variations on the expression of inflammation-related factors in the differentiated cardiomyoblasts. The low-frequency (0.25 Hz)/low-elongation (2%) stretching protocol was found to significantly downregulate the expression of NF-kB and IL-6 and not upregulate the expression of TNF-α (Figure 6A,B,D). Moreover, low-frequency stretching appeared to decrease the expression of IL-6 independently of the magnitude of elongation (Figure 6B). In addition, it was shown that mechanical stretching induces the upregulation of IL-1β regardless of the stretching characteristics (Figure 6C).

#### 3.2.5. Changes in the Activation of Key Intracellular Signalling Mediators

**Figure 7:**
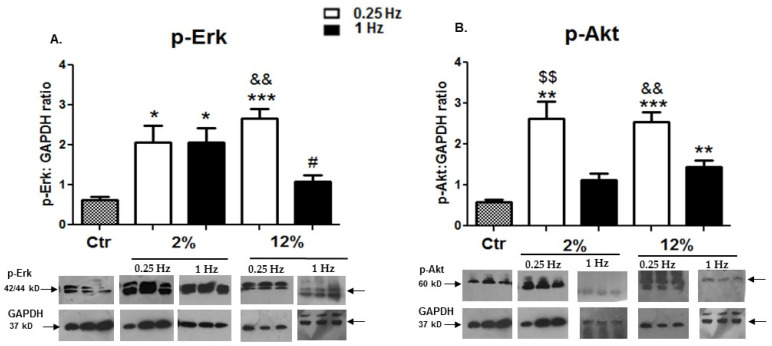
(**A**,**B**) Effects of cyclic mechanical stretching on the activation of the signalling proteins Akt and Erk1/2. Representative Western blots and immunoblotting quantification of (**A**) p-Erk1/2 and (**B**) p-Akt phosphorylation in cardiomyoblasts subjected to different mechanical loading protocols compared to the control (Ctr; nonstretched myotubes). The values of the phosphorylated proteins were normalized to each corresponding GAPDH on the same immunoblot. The same blots have been used for GAPDH in Figure 3D,E, in Figure 5C, and in Figure 7A,B except for the blot of GAPDH in (**A**) for the 2%/1 Hz stretching protocol. Significantly different compared to the control * *p* < 0.05; ** *p* < 0.01; *** *p* < 0.001. Significantly different compared to the 2%/0.25 Hz stretching protocol, ^#^ *p* < 0.05. Significantly different compared to the 2%/1 Hz stretching protocol, ^$$^ *p* < 0.01. Significantly different compared to the 12%/1 Hz stretching protocol, ^&&^ *p* < 0.01. Mean + SE of three independent experiments performed in triplicate.

The effects of different loading protocols on the activation of important signalling proteins, associated with cell growth and survival pathways, were also investigated in the H9C2 cardiomyotubes. We found that all stretching protocols used resulted in higher levels of Erk 1/2 phosphorylation compared to the control condition (Figure 7A). In addition, mechanical stretching induced, overall, a higher activation of the Akt pathway compared to the control, while the lower-frequency (0.25 Hz) stretching protocols, particularly, appeared to cause higher phosphorylation levels of Akt compared to high-frequency mechanical loading (Figure 7B).

## 4. Discussion

This study investigated cellular and molecular events during the differentiation of cardiomyocyte-like H9C2 cells, and compared the effects of various mechanical stretching protocols on signalling and gene expression responses associated with the myogenic lineage and survival program of the differentiated cells. Based on our previous work [29,37], we hypothesized that the molecular responses elicited by these cells following mechanical loading would vary depending on the loading characteristics of the protocols used, revealing the specific detrimental or beneficial effects on the differentiated cardiomyocytes. The main findings of this study were that H9C2 cells possess a myogenic differentiation potential towards a cardiac-like gene expression phenotype, eventually forming multinucleated cardiomyotubes, while altering the strain and frequency of mechanical loading applied on the cardiomyotubes can regulate their anabolic/survival program. Specifically, a main finding of particular interest was that a low-strain/frequency stretching was, overall, the most effective at inducing protein synthesis and myogenic lineage along with the suppression of apoptosis, inflammation, and atrophy in these mechanosensitive cells.

Myogenic differentiation is a highly orchestrated process driven by multiple signal transduction and mechanotransduction pathways, which coordinate the balance between muscle growth and atrophy [24,36,39,40,41]. Cell cycle arrest and morphological alterations appear to accompany the differentiation process of the cardiomyocyte-like H9C2 cells [3]. Following on from previous reports [2,3], the present study further characterized the ability of these cells to exhibit a cardiac myogenic differentiation, as demonstrated by the time course expression of myogenic transcription factors (MRFs) and cardiac-specific markers, as well as by the morphological analyses of these cells during the differentiation process (Figure 1). More specifically, undifferentiated H9C2 cells are mononucleated, exhibiting a normal cell cycle progression; however, their differentiation progressively leads to their fusion into multinucleated cardiomyotubes. This potential was documented by the gradual increase in their fusion and maturation indexes, and the increased expression of the myogenic and cardiac differentiation markers MyHC, actin, and troponin. These alterations were accompanied by the downregulation of the cell cycle regulatory protein Cyclin D1 and the reduced activation of ERK1/2 pathway, attesting to the suppression of proliferation rate of these cells during differentiation (Figure 1B,G–K). In addition, we found that the progression of H9C2 cardiomyoblast differentiation was also accompanied by a gradual increase in p38 activation over time, which has been shown to inhibit the activation of ERK1/2 and to induce cell cycle arrest in adult mammalian cardiomyocytes [42]. Furthermore, and in accordance with previous studies, our study documented the increased phosphorylation of Akt in the differentiating H9C2 cells, which is an important mediator of protein synthesis, a critical process for the completion of myogenic differentiation [40,41] (Figure 2C–E).

Indeed, myogenic differentiation is associated with the induction of anabolic processes and increased protein synthesis [43], while MRFs and growth factors function as activators and/or mediators of muscle cell differentiation [22]. In particular, interactions between MRFs and growth factors, such as IGF-1, have been previously described in skeletal muscle cells [24,44,45] and this study demonstrated a coordinated differential expression of the early (MyoD) versus late (MRF4) myogenic regulators over time in the differentiating H9C2 cardiomyocyte-like cells, while IGF-1 isoforms also exhibited a different regulation during the differentiation process of the cardiomyoblasts. It has been documented that IGF-1 is a key factor involved in myogenesis and is a major regulator of cardiomyocytes development and growth [25,26]; however, to the best of our knowledge, this is the first study examining the distinct expression profiles of IGF-1 isoforms in H9C2 cardiomyoblasts during their differentiation. Specifically, our data showed a persistent upregulation of the IGF-IEa isoform throughout the differentiation process, along with the downregulation of the IGF-1Eb at the advanced stage of differentiation, further supporting the notion of a differential regulation and role of IGF-1 isoforms in muscle biology [28,38,44,46,47,48] and in myocardial remodelling [26,45], but also in other biological systems [49,50,51,52,53,54].

This study further investigated the myogenic and anabolic potential of differentiated H9C2 cells following mechanical loading, since their ability to convert external mechanical stimuli into biochemical signals is critical for the maintenance of their homeostasis and for the adaptation of cardiac muscle to mechanical loading [4,5,55]. Our previous work has shown that different effects on the myogenic lineage of advanced differentiation cardiomyoblasts are elicited by varying the characteristics (i.e., strain, frequency, duration) of mechanical stretching applied on them, indicating specific features of loading for regulating the anabolic/survival program in these cells [29]. Specifically, it was found that, regarding the duration of mechanical loading, an intermediate-duration (12 h) stretching protocol was, overall, the most effective at inducing beneficial responses in the stretched cardiomyoblasts. Thus, in the present study we utilized this “optimum” duration of loading to further explore the effects of strain/frequency variations on myogenic, hypertrophic, anti-apoptotic, and anti-inflammatory responses, and on the activation of major intracellular signalling cascades in cardiomyotubes.

Myogenic differentiation of myoblasts is regulated by MRFs, and while studies have shown that mechanical stimuli affect the expression of these myogenic determinants [56], the responses of MRFs to mechanical loading in cardiomyocytes remain largely unknown [29]. In the new series of loading protocols used in this study, we further revealed that at the “optimum” duration the high-frequency (1 Hz) mechanical loading applied on the differentiated cardiomyocytes may induce lower expression of the myogenic determinants MyoD, Myogenin, and MRF4 compared to low-frequency (0.25 Hz) stretching protocols, regardless of their strain features (Figure 3). Moreover, our findings suggest that these MRFs, at least at this late stage of myogenic differentiation, are responsive to mechanical loading and more sensitive, particularly to low-frequency stretching. Interestingly, this study showed that MRF4 appears to be responsive only to low strain loading, potentially indicating a differential responsiveness of this late myogenic factor to mechanical stimuli due to its distinct role(s) in that stage of the myogenic differentiation program [37].

Mechanical loading of skeletal and cardiac muscle cells can induce the upregulation of many growth factors associated with protein synthesis and cell growth, eventually leading to muscle hypertrophy [27,30,44,57,58]. In particular, the upregulation of IGF-1 has been implicated in the adaptive cardiac hypertrophy induced by mechanical loading, while potentially differential actions of IGF-1 isoforms in the myocardial repair/remodelling process have been proposed [26,59]. Our findings showed that both isoforms were upregulated by low-frequency stretching protocols, while strain appeared to regulate the magnitude of overexpression, with more pronounced increases being exhibited after the high-strain/low-frequency protocol and vice versa.

We further explored the transcriptional responses of skeletal and cardiac muscle atrophy genes Atrogin-1 and Myostatin to the various mechanical stimuli applied on cardiomyoblasts, since both are negatively involved in myogenesis, acting through the ubiquitin–proteasome pathway [31,55,60,61] and actively inhibiting protein synthesis [57,58,62]. Interestingly, we found that, compatibly with the anabolic upregulation of IGF-1 isoforms by low-strain/frequency loading shown in this study, the same protocol was also the only one that did not upregulate those atrophy genes, suggesting that such mechanical loading features may effectively regulate the anabolic program in the differentiated cardiomyoblasts.

Various pro-apoptotic factors may be involved in myogenic differentiation, like FoxO, which seems to be a fate decider within the myogenic program [63] while also inhibiting Akt and Erk1/2 activation, inducing cardiac muscle wasting, and promoting heart failure [33]. Our previous work has shown that FoxO1 is responsive to mechanical loading [29] and the present study further revealed that its expression pattern was similar to that of muscle atrophy factors. In particular, the low-strain/frequency protocol was again the only one that did not induce FoxO1 upregulation, while, as also observed for the atrophy genes, increasing the elongation of stretching at the same low frequency resulted in the upregulation of FoxO1 (Figure 4 and Figure 5). However, different expression responses to the various loading protocols were found for p53, a pro-apoptotic factor that has been proposed to suppress muscle differentiation at the Myogenin step [64]. Indeed, p53 exhibited a reverse expression pattern, both at the transcriptional and translational level, compared with Myogenin responses to the various loading protocols tested (Figure 3 and Figure 5). Our findings suggested that the potential regulatory interactions between p53 and Myogenin within the myogenic differentiation of cardiomyoblasts may be mechanical loading dependent.

Moreover, p53 and its downstream effectors have been proposed to promote an inflammatory cytokine-mediated inhibition of myogenic differentiation [64]. Thus, although many studies have suggested beneficial effects of mechanical stimuli on cardiomyocytes structure and function, nevertheless excessive mechanical loading have been reported to induce cardiac cell apoptosis and the upregulation of muscle atrophy and pro-inflammatory factors [65,66]. Therefore, to further associate mechanical stimuli with the pathophysiology of cardiomyopathies, this study investigated the effects of mechanical loading variations on the expression of inflammation-related factors in the differentiated cardiomyoblasts. Our data showed that, again, the low-strain/frequency stretching protocol resulted in decreased expression of the inflammation-related factors NF-kB and IL-6, while increasing the strain and/or the frequency of stretching was found to increase the expression responses of IL-1β, TNF-α, NF-kB, and IL-6. These findings implied a loading-induced increase of the inflammatory potential in cardiomyotubes, depending on the specific features of mechanical loading. Overall, the findings above indicate a multiple beneficial effect of low-strain/frequency, intermediate-duration (12 h) stretching, as it simultaneously upregulates the myogenic/anabolic program and downregulates atrophy, apoptotic, and inflammatory factors in advanced-differentiation cardiomyocytes.

Lastly, two primary mechanosensitive intracellular pathways appear to require comprehensive identification of their interactions and outcomes within the context of cardiac and myogenic differentiation and their pathophysiological transfer to cardiomyopathy under mechanical stimuli [27,30,31,44]. Specifically, the activation of the phosphatidylinositol 3-kinase (PI3K)/Akt pathway has been implicated in myocardial cells’ survival and their protection against reperfusion-induced injury and apoptosis, while the Ras/Raf/Erk1/2 signalling pathway has been shown to be essential for myocardial hypertrophy [25]. Our previous work has shown a loading-specific activation of these two pathways in differentiated cardiomyocytes in vitro, with the intermediate-duration (12 h) stretching protocol being effective at inducing upregulation of both Erk1/2 and Akt pathways [29]. In the present study, we used this “optimum” intermediate duration of loading to further investigate the effects of frequency and strain variations on the activation of these major signalling pathways. Our new data further revealed that low-frequency loading of cardiomyoblasts may be, overall, more effective at the activation of the signalling mediators Erk1/2 and Akt compared to the high-frequency protocols, regardless of the strain features (Figure 7). These findings suggest that the loading-induced activation of these pathways is not mutually exclusive and may depend on the characteristics of mechanical loading.

## 5. Conclusions

The in vivo models of loading-induced cardiac adaptations are quite complex and, thus, in vitro models of mechanical loading applied on myocardial cells are crucial for understanding the cellular and molecular mechanisms that mediate loading-induced adaptations. This study further characterized the differentiation process of H9C2 cells towards a cardiac-like phenotype and utilized advanced-differentiation cardiomyocytes in an in vitro model of cell stretching to investigate intracellular molecular events induced by mechanical stimuli. By varying specific characteristics of mechanical loading, we have advanced on previous findings and emphasized the optimal characteristics of mechanical loading in terms of its strain and frequency features for eliciting predominantly beneficial adaptations in the loaded cardiomyotubes. These findings may be a useful resource in translational research for the development of more targeted experimental approaches to mimic adaptive remodelling processes that are possibly driven by mechanotransduction, such as the exercise-induced physiological cardiac hypertrophy or the maladaptive remodelling of the myocardium. Indeed, pathological hypertrophy-, apoptosis-, atrophy-, and inflammation-related responses to mechanical stimuli investigated in this study characterize serious cardiac disorders, such as myocardial infarction, hypertension-induced left ventricular hypertrophy, dilated cardiomyopathy, and heart failure. Elucidation of such novel mechanisms might provide novel therapeutic targets and help us develop future pharmacotherapies to treat mechanical loading-associated cardiomyopathies.

## Figures and Tables

**Table 1:** The sequences of the specific sets of primers used for RT-PCR analyses.

Target Gene	5′-3′ (Forward) Primer Sequence	5′-3′ (Reverse) Primer Sequence
GAPDH	CAA CTC CCT CAA GAT TGT CAG CAA	GGC ATG GAC TGT GGT CAT GA
MYOD	TGC TCC TTT GAG ACA GCA GA	AGT AGG GAA GTG TGC GTG CT
MYOGENIN	AGG AGA GAA AGA TGG AGT CCA GAG	TAA CAA AAG AAG TCA CCC CAA GAG
MRF4	AGG GCT CTC CTT TGT ATC CAG	TGG AAG AAA GGC GCT GAA GA
cTnT	GCG GAA GAG TGG GAA GAG ACA	CCA CAG CTC CTT GGC CTT CT
CYCLIN D1	TCA AGT GTG ACC CGG ACT G	ATG TCC ACA TCT CGC ACG TC
IGF-1Ea	GTG GAC GCT CTT CAG TTC GT	GCT TCC TTT TCT TGT GTG TCG ATA G
IGF-1Eb	GTC CCC AGC ACA CAT CGC G	TCT TTT GTG CAA AAT AAG GCG TA
p53	GAG AGA CCG CCG TAC AGA AG	AGC AGT TTG GGC TTT CCT CC
FoxO1	AGT GGA TGG TGA AGA GCG TG	GAA GGG ACA GAT TGT GGC GA
TNF-α	CTC TTC TGC CTG CTG CAG TTG	ATG GGC TAC AGG CTT GTC ACT C
NF-kB	ATA GGC ACT GTC TTC TTT CAC CTC	ATA GGC ACT GTC TTC TTT CAC CTC
IL-6	CCT TCC TAC CCC AAT TTC CAA T	AAC GCA CTA GGT TTG CCG AGT A
IL-1β	ATC CCA AGC AAT ACC CAA AG	GTG CTG ATG TAC CAG TTG GG
ATROGIN-1	AAC AAG GAG GTA TAC AGT AAG G	AAT TGT TCA TGA AGT TCT TTT G
MYOSTATIN	CTG TAA CCT TCC CAG GAC CA	GCA GTC AAG CCC AAA GTC TC

## Data Availability

Not applicable.
